# Common Multiple Primary Cancers Associated With Breast and Gynecologic Cancers and Their Risk Factors, Pathogenesis, Treatment and Prognosis: A Review

**DOI:** 10.3389/fonc.2022.840431

**Published:** 2022-06-08

**Authors:** Shuwen Ge, Bo Wang, Zihao Wang, Junjian He, Xiaoxin Ma

**Affiliations:** ^1^ Department of Obstetrics and Gynecology, Shengjing Hospital of China Medical University, Shenyang, China; ^2^ Key Laboratory of Maternal-Fetal Medicine of Liaoning Province, Key Laboratory of Obstetrics and Gynecology of Higher Education of Liaoning Province, Shenyang, China

**Keywords:** breast cancer, ovarian cancer, endometrial cancer, cervical cancer, multiple primary cancers, primary gynecologic cancer

## Abstract

The mammary gland is closely related to the female reproductive system in many aspects, affecting the whole gynecological system. Breast cancer (BC) is the most common malignancy in women and associated with considerable negative effects. Due to various factors including co-pathogenic genetic mutations, environment factors, lifestyle, behavioral factors, treatment regimens and in-creased survival of patients with BC, there is an increased probability of developing additional primary gynecologic cancers such as ovarian cancer (OC), endometrial cancer (EC), and cervical cancer (CC). More and more studies have been conducted in recent years. Multiple primary cancers (MPCs), also known as multiple primary malignancies, refers to two or more different primary cancers in the same patient occurring in the same or different organs or tissues. The pathogenesis of multiple primary cancers is complex and has a negative effect on the prognosis and survival of patients. This review discusses the common types of BC-associated MPCs, namely, BC associated with OC, BC associated with EC and BC associated with CC, as well as risk factors, pathogenesis, treatment, and prognosis of MPCs associated with breast and gynecologic cancers. It provides new intervention and treatment ideas for patients with BC-associated MPCs to improve quality of life and prognosis.

## Introduction

Breast cancer (BC) is the most common malignancy in women and is associated with considerable negative effects (1–6). Its 5-year incidence is approximately 43.8 million cases worldwide (7). Despite its increasing incidence in recent years (8), the mortality rate of women with BC has been decreasing (9, 10) due to early screening and continuous improvement in treatment (11).

Multiple primary cancers (MPCs), also known as multiple primary malignancies, refer to two or more different primary cancers in the same patient occurring in the same or different organs or tissues (12). The most common sites are paired organs, especially the breasts, gastrointestinal tract, and thyroid gland (13). Double primary cancer is the most common type of MPCs (14), whereas the incidence of quadruple primary cancer is only <0.1% (15).

**Figure 1:**
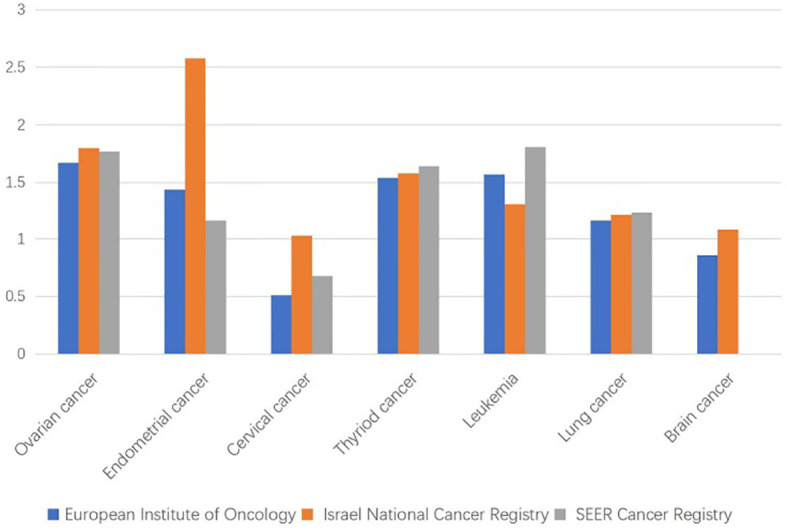
Standardized incidence ratios for common multiple primary cancer sites associated with breast cancer (BC).

The incidence of MPCs associated with BC is also increasing (16) due to increased patient survival (17), genetic susceptibility, environmental interactions, as well as chemotherapy, seriously affecting the quality of life and prognosis of patients. The incidence of a secondary primary cancer after BC is between 4% and 16% (18), which is approximately 17% higher than in healthy women (19), with the most common types being thyroid cancer, gynecological malignancy, etc. (16, 20, 21) **(** **Figure 1**). As shown in **Figure 1**, the blue (22) represents a cohort study of 21, 527 patients with BC at cancer centers in Italy from the European Institute of Oncology. The orange (20) represents a cohort study of patients with BC and other primary cancers in Israel National Cancer Registry between 1992 and 2006. The data does not include follow-up information after 6 months of BC diagnosis. The gray (23) represents patients aged 50 to 59 years with BC and other primary cancers in the SEER Cancer Registry. The data on brain cancer are not available. It can be inferred that the most common gynecologic cancers among the BC-associated MPCs include ovarian cancer(OC), endometrial cancer(EC) and cervical cancer(CC).

The mammary glands are closely related to the female reproductive system; therefore, gynecologic malignancies are also the most common primary cancer after BC (24). Multiple studies have shown that among the MPCs associated with BC, female reproductive malignancies such as OC, EC, and CC are the most common malignancies (25–28), which is consistent with the inference mentioned in the previous paragraph.

In recent years, although evidence about the pathogenesis, treatment, prognosis, and survival in BC associated with gynecologic malignancies has increased, there is still a need for a comprehensive review. In this review, we discuss three common types of MPCs associated with BC, namely, BC associated with OC, EC, and CC, as well as risk factors, pathogenesis, treatment, and prognosis of MPCs associated with breast and gynecologic cancers. The aim is to provide new intervention and treatment ideas for patients with MPCs associated with breast and gynecological malignancies to improve quality of life and prognosis.

## MPCs Associated With BC and OC

Early studies have shown that BC is associated with an increased risk of developing OC (29) and that OC is the most common primary malignancy after BC (30). Recently, Nasioudis et al. found that the incidence of BC increases after the incidence of ovarian granulosa cell tumor (31). The risk factors for MPCs are mostly genetic (32). In 1994, Ford et al. established the fact that the carriers of the *BRCA-1* mutation are more likely to develop BC and OC (33), and mutations in the BRCA genes are generally linked to BC and OC (34–36). Through large-scale sequencing, Lu et al. recently identified genes including *MSH6* and *ATM* as genes that increase an individual’s susceptibility to BC and OC (37). In the same year, Samadder et al. found that a disease-causing mutation in *PALB2* was associated with a medium-high risk of developing BC and OC (38). *CDKN2A* mutations have also been detected in both cancers (39). Furthermore, Peutz–Jeghers syndrome, which is caused by *STK11* mutation, results in malignant changes in the mammary glands and ovaries (13). According to a family-based study conducted in 2020, variations in *RAD51C* and *RAD52D* are correlated with an increased risk of developing BC and OC (40). In conclusion, the mutation of some co-pathogenic genes associated with the breasts and ovaries can induce MPCs in these organs.

Regarding the common clinicopathological classification of BC associated with primary OC, studies have reported that BC and epithelial OC have similar risk factors (41). Therefore, patients with one type of cancer have an increased risk of developing other cancers in future. Studies have shown that patients with *BRCA1* and *BRCA2* mutations are susceptible to developing BC associated with OC, and the probability of developing epithelial-type BC is higher, as is that of developing the histological subtype of serous OC (38, 42–44). Regarding molecular typing, *BRCA1*-associated BC is of the medullary subtype with a high proportion of negative estrogen and progesterone receptor expression (43, 45). *BRCA2*-associated BC is similar to luminal-type tumors. Patients with triple-negative BC are more likely to develop primary OC (22). In addition, Bryk et al. reported that the standardized incidence of primary BC in patients with adult granulosa cell tumors (AGCTs) was 1.26 (95% CI: 0.92–1.73) and that of AGCTs after BC was 1.59 (95% CI: 1.04–2.29) (46). Therefore, the prevention and treatment of BC should be considered in the follow-up examination along with identification of patients with AGCT.

Studies show that BC is most likely to develop in the first few years after OC diagnosis (47), and after 10 years, the risk of developing BC is 7.8% (48). Additionally, OC is the most common primary cancer after BC (30). Therefore, regardless of the primary origin, patients with hereditary breast and ovarian cancer should undergo regular screening in the first few years after diagnosis so that timely intervention and treatment are provided to improve prognosis and quality of life (49). Several studies have reported that mastectomy after OC in patients with *BRCA* mutations reduces the risk of developing BC (45, 48, 50). Thus, prophylactic mastectomy, which reduces the risk of developing BC by 90%, should be considered for *BRCA1/2* mutation carriers to improve prognosis (50). In addition to the cancer antigen 125 test (50) performed every 6 months, use of oral contraceptives as well as prophylactic salpingectomy and oophorectomy can lower the risk of developing OC (38, 51, 52). Moreover, prophylactic salpingo-oophorectomy is associated with a reduced risk of developing BC (53).

Regarding treatment type, studies have shown that patients with *BRCA* mutations who are treated with cisplatin have higher complete response rates than those treated with other chemotherapy regimens (54). Moreover, in recent years, studies have shown that *BRAC1* mutations are associated with an increased expression of PD-L1 and PD-1 (55); this suggests that checkpoint inhibitors have an effect on *BRCA*-associated cancers (43), thereby providing a new treatment approach for MPCs associated with BC and OC.

In conclusion, screening should be regularly performed for patients with genetic mutations who are susceptible to developing primary BC and OC, and prophylactic mastectomy/salpingo-oophorectomy and drug therapy should be considered for risk reduction.

## MPCs Associated With BC and EC

EC is a common secondary primary cancer among patients with BC (56), and the causes of this combination of MPCs are complex. In terms of the common pathogenic genes, Guo et al. recently reported that mutations of *p53*, *HER-2*, and other genes are related to the incidence of both BC and EC (57). Kim et al. found that *IMP3*, which is associated with triple-negative BC, is also related to type II EC (58). In addition, Cowden syndrome (also known as multiple hamartoma syndrome) is often associated with thyroid cancer, BC, and EC and is caused by a *PTEN* mutation, which also increases the risk of developing EC in patients with BC (59), making them susceptible to MPCs (60). Studies have confirmed that the long non-coding RNA, NR2F1-AS1, can promote BC and EC progression (61), becoming a co-pathogenic factor for both types of cancer, and is possibly one of the causes of BC associated with primary EC.

Obesity-related diseases can promote the occurrence and development of BC and EC. It is well known that metabolic syndrome is closely related to EC (62), considering that most patients with metabolic syndrome show signs of obesity. The presence of aromatase in the excessive adipose tissue of patients leads to the transformation of androgens into estrogens (63), resulting in the rise of estrogens in patients; abnormal estrogen levels are also driving factors leading to BC (64). In addition to its effects on estrogen levels, metabolic syndrome can also lead to hyperinsulinemia (65), which can increase the level of insulin-like growth factor-1 (IGF-1). IGF-1 expression was increased in both BC and EC cells (66, 67). The possible mechanism is that the overexpression of insulin growth factor receptor-1R (IGF-1R) or insulin receptor (IR) leads to mitotic signaling and increases the activation of phosphoinositide-3-kinase (PI3)-Akt-mTOR signaling pathway (68), which mediates the development of BC and EC. In addition, insulin resistance and hyperinsulinemia in type II diabetes mellitus patients lead to an increased incidence of BC and EC (69). Therefore, metabolic disorders of obesity-related diseases or a variety of hybrid effects of obesity are possible factors influencing the pathogenesis of MPCs associated with BC and EC.

BC treatment can also affect the development of EC. Tamoxifen, the most commonly used endocrine therapy for patients with hormone receptor-positive BC (70–72), acts as an estrogen antagonist in the breasts and as an agonist in the uterus (73). Therefore, the use of tamoxifen is closely related to the development of EC (74–77). A study in Denmark showed that the relative risk of developing EC in patients with BC who were treated with tamoxifen was 1.5 (78), and the risk increases significantly with the duration of drug use (79). A study claimed that 70.7% of the patients with BC will be diagnosed with EC within 5 years (57). Tamoxifen increases the risk of developing EC by 2–7 fold, and aggressive EC types with poor prognosis have been observed among tamoxifen users (75). The long-term use of tamoxifen resulted in a 59% higher risk of EC-related death in women who had received it for 5 years or more than in women who had not (80). Therefore, timely baseline tamoxifen screening should be performed in these patients (81) through regular transvaginal ultrasound, endometrial examination, and biopsy (82) to lower the threshold (83), diagnose EC, and detect malignant endometrial changes in a timely manner. Because tamoxifen use can lead to poor outcomes, Chlebowski et al. reported that aromatase inhibitors can reduce the incidence of tamoxifen-related EC (84), and they are effective against tamoxifen-resistant BC due to a different mechanism of action (85).

However, genomic analysis showed that tamoxifen-induced endometrial tumors were not different from those in patients who did not receive tamoxifen (86), and other studies have found that patients with ER(−) PR(−) BC who are the least likely to receive tamoxifen also have a significantly increased risk of developing EC (83). This suggests that tamoxifen is not the only risk factor for EC. In addition to genetic mutations and treatment factors, increased body mass index is associated with an increased risk of developing EC in patients with BC (87, 88).

Uzunlulu et al. reported that the incidence of EC in patients with both ER(+) and HR(−) BC was higher than that in the general population and that the clinicopathological characteristics of both are similar (89). This is consistent with the results reported by Guo et al. who found that the incidence of ER(+) and HR(−) BC associated with EC was 0.33% and 0.30%, respectively, which were approximately 16 and 15 times higher than that in the general population (57). Further, EC after BC was a common MPC, and there were no significant differences in the stage or distribution of pelvic or para-aortic metastases compared with those in patients with primary EC alone (57). The effect of the hormone receptor status on the endometrium in patients with BC requires further investigation.

## MPCs Associated With BC and CC

CC accounts for 13.4% of all MPCs. The incidence of CC-associated MPC is increasing, most commonly coexisting with BC, lung cancer, and colorectal cancer (90). The development of CC-associated BC is closely related to human papillomavirus (HPV) infection. The high expression of P16, an indicator of HPV transcriptional activity, increases in malignant breast tissues (91). A case study reported that the same high-risk HPV types with biological activity were found in the breast and cervical tissues of some patients with BC or CC, with HPV18 being the most common type (92). The incidence of high-risk HPV infection in BC is four times higher than that of benign BC (93). suggesting that women with HPV infection-associated CC are also at a risk of developing BC. Patients with surgically treated high-grade cervical intraepithelial neoplasia (CIN 2/3) also have an increased risk of developing HPV infection-associated BC and OC (94). In addition to the independent role of HPV, recent studies have found that it may co-infect with Epstein–Barr virus (EBV) to promote the occurrence and progression of CC, BC, as well as other cancers (95), and co-infection of high-risk HPV and EBV possibly plays an important role in the progression of BC through the ERK1/ERK2 and β-catenin signaling pathways (96). Long-term oral contraceptive use also increases the risk of BC and CC in HPV-infected women (97). Therefore, patients with HPV infection who need to take contraceptives over a long period of time should be made aware of this risk. In particularly, BC patients with HPV infection should be vigilant for CC during treatment.

Moreover, it has been reported that PIN1, an enzyme that changes the conformation of phosphoprotein, is overexpressed in malignant tumors such as BC and CC (98), and that patients with PIN1 overexpression are likely to develop MPCs; such patients should be screened for disease in time. In addition, the treatment of BC patients can also cause a second primary cancer. Thong et al. found that alkylating agents in combination with chemotherapy can lead to the development of malignancies (99), which is consistent with the findings of Hughes et al. who discovered that the use of alkylating agents in combination with chemotherapy in patients with BC contributed to the development of CC (100).

There are few reports on the clinical classification and characteristics of CC-associated BC. As for prognostic markers, it has been reported that the expression level of FGD3 mRNA has prognostic value in BC and cervical squamous cell carcinoma (101).

Since BC accounts for a certain proportion of patients with CC, regular follow-up and detection should be performed on the mammary glands and female reproductive organs of patients with CC.

## Risk Factors

In recent years, with the increased survival of cancer patients, the incidence of BC-associated MPCs has also been increasing. And is often caused by common risk factors such as genetic and environmental factors, lifestyle and treatment regimens (102, 103). The prognosis has also been reported and common drugs used to treat these cancers could provide new insights into the treatment of BC-associated MPCs. The following are common risk factors.

### Genetic Factors

The occurrence and development of tumor is closely related to DNA damage. The presence of co-pathogenic genetic mutations is likely to lead to the occurrence of MPCs associated with breast and gynecologic cancers. The list of genetic mutations and associated syndromes in MPCs associated with breast and gynecologic cancers is shown in **Table 1**. In addition, a study reported that microsatellite instability is more common in MPCs than in sporadic cancers (104), demonstrating its likely influence on the pathogenesis of MPCs.

**Table 1:** List of genetic mutations and associated syndromes in multiple primary malignancies of breast and gynecologic cancers.

Disease	Gene	Associated syndrome
*BC and OC*	*BRCA1/BRCA2*	Hereditary breast and ovarian cancer (13)
	*MSH6*	Lynch syndrome (13)
	*ATM*	Ataxia–telangiectasia (35)
	*PALB2*	Fanconi anemia (35)
	*CDKN2A*	Familial atypical multiple mole melanoma syndrome (13)
	*STK11*	Peutz–Jeghers syndrome (13)
	*RAD51C/RAD51D*	–
*BC and EC*	*p53*	–
	*HER-2*	–
	*PTEN*	Cowden syndrome (13)
	*IMP3*	–
*BC, EC and* *OC*	*MLH1, MSH2*, *MSH6*	Lynch syndrome (13)

### Estrogen Factor

Because the mammary gland, uterus, and ovaries have the same estrogen receptors, these areas are simultaneously affected by estrogen-related disturbances. BC is associated with an increased risk of the development of other female reproductive system tumors. Studies have shown that abnormal estrogen levels are closely correlated with BC, EC, OC, and CC in women and are risk factors for many other cancers (85, 105, 106). In addition to abnormal estrogen levels, abnormal estrogen receptor signaling can also contribute to breast, endometrial, and ovarian cancers (106).

Estrogen can lead to different molecular types of BC. One study showed that patients with luminal BC were more likely to develop heterogeneous EC or OC (107), and luminal BC has a strong association with estrogen. If the developmental trend and prognosis of MPCs can be determined by molecular typing of BC, it can provide a clear and efficient treatment plan for these patients.

### Lifestyle and Behavioral Factors

Lifestyle and behavioral factors considerably influence the occurrence, development, and treatment of BC-associated MPCs. A high-fat diet can lead to fluctuations in estrogen levels, which can, in turn, lead to the development of OC, BC, and EC (108). Many studies have shown that overweight and obese patients have a higher risk of cancer recurrence (109, 110). For example, most patients with type I EC have metabolic syndrome, which is also associated with BC (111), and bariatric surgery can reduce the risk of developing not only BC but also EC and OC (112). Studies have shown that aromatase in adipose tissue can lead to high estrogen levels, inducing hormone-related cancers such as BC, EC, and OC (113). Adipose tissue is also rich in hormones, cytokines, and other mediators such as leptin and adiponectin, giving rise to a microenvironment that promotes cell proliferation and inflammation (106). Leptin-induced cell signaling cascaded to increase the risk of BC, EC, and OC (114). Therefore, in daily life and in the course of cancer treatment, strict weight management and control should be carried out to maintain the normal metabolism of the body and the normal level of hormones in body, so as to prevent the occurrence of MPCs associated with BC and gynecological cancers.

In addition, smoking is a risk factor contributing to the development of secondary primary cancer (115), and its synergistic relationship with treatment may be associated with the highest risk of secondary primary cancer (109). Quitting smoking after an initial cancer diagnosis may delay the development of secondary primary cancer (116).

### Age

In addition to genetic mutations, hormone receptor status, lifestyle and behavioral factors, early age at first diagnosis of BC, and race were also found to be risk factors for primary gynecologic malignancy after BC (117). Young women are more likely to suffer a second primary cancer than women over the age of 50 (118), and second primary cancers of the female reproductive system after BC are also more likely happen to younger women (119). The 10-year survival rate of women aged 20–29 years with BC-associated MPC is approximately 50% lower than that for women with BC alone (23). Therefore, the treatment of these young patients needs additional attention and research.

### Treatment Regimens

Treatment is a double-edged sword, it can play a therapeutic role in the first primary cancer, but also can pave the way for the second primary cancer. For example, as mentioned above, tamoxifen, which is used to treat BC, can also cause EC. Otherwise, radiotherapy can also significantly increase the incidence of other primary cancers (109, 120). Berrington de Gonzalez and his team have shown that about 3% of secondary primary cancers in BC survivors are caused by radiation therapy (121), and Grantzau and Overgaard confirmed that patients who have received radiation therapy for BC were 23% more likely to develop another primary cancer (122). Patients with BC who underwent chemotherapy also had a higher standardized incidence of gynecologic malignancies than those who did not undergo chemotherapy (110).

## Pathogenesis

To sum up, when the mammary gland and the female reproductive system are exposed to a certain intensity of carcinogenic factors (such as abnormal estrogen levels, terrible lifestyle and behavior patterns, etc.) for a long time, extensive tissue and cells will appear DNA damage, local regional abnormalities and even precancerous lesions (123). The DNA of these cells will be further altered, including oncogene activation and tumor suppressor gene inactivation, by the combined effects of continued oncogenic action or treatment of primary cancer (124) (chemoradiotherapy, hormonal drugs such as tamoxifen, etc.). If the patient has the pathogenic genes associated with BC and gynecologic cancers, the patient is more likely to develop secondary malignant tumors in the final stage, thus forming MPCs with independent and different clonal sources. This indicates that secondary tumors are caused by the human microenvironment similar to that of the first primary cancer, or are closely related to the treatment of the first primary cancer. Simultaneous MPCs can affect the treatment of the first primary cancer in patients. The interaction mechanism between MPCs is complex and diverse, which still needs further research and exploration.

## Treatment

BC-associated MPCs is often caused by common risk factors. Therefore, finding the common pathogenic factors and co-therapeutic drugs of these cancers may be a key breakthrough in the treatment of MPCs associated with breast and gynecological cancers. Patients with BC or gynecological cancers should be targeted at the above risk factors for prevention or treatment. Once the second primary cancer is found, radical measures should be taken immediately to intervene in the progression of the disease.

For genetic factors, these patients should be screened for genetic screening, so as to carry out targeted prevention of subsequent cancers. The status of the breasts and reproductive system should be closely monitored during the treatment of patients with BC or gynecologic malignancies, corresponding preventive resection should be carried out when necessary, and radiotherapy or chemotherapy should be carried out according to the condition.

For abnormal estrogen levels, anti-estrogen receptor drugs are widely used to treat these diseases. In recent years, studies have shown that fulvestrant, a selective estrogen receptor degrader, has a strong therapeutic effect in both BC and EC models *in vitro* and *in vivo* (125). The use of selective estrogen receptor modulators and selective estrogen receptor degraders to treat MPCs associated with breast and gynecological cancers is undoubtedly a way to improve the prognosis. In addition, studies have found that pigment epithelial derived factor (PEDF) can inhibit the occurrence of tumors in the mammary gland, ovary, endometrium, and other places through the negative regulation of estrogen. PEDF inhibits cell proliferation and reduces cell invasion, and may also prevent drug resistance of BC, EC, and OC by down-regulating estrogen receptors (126).

**Figure 2:**
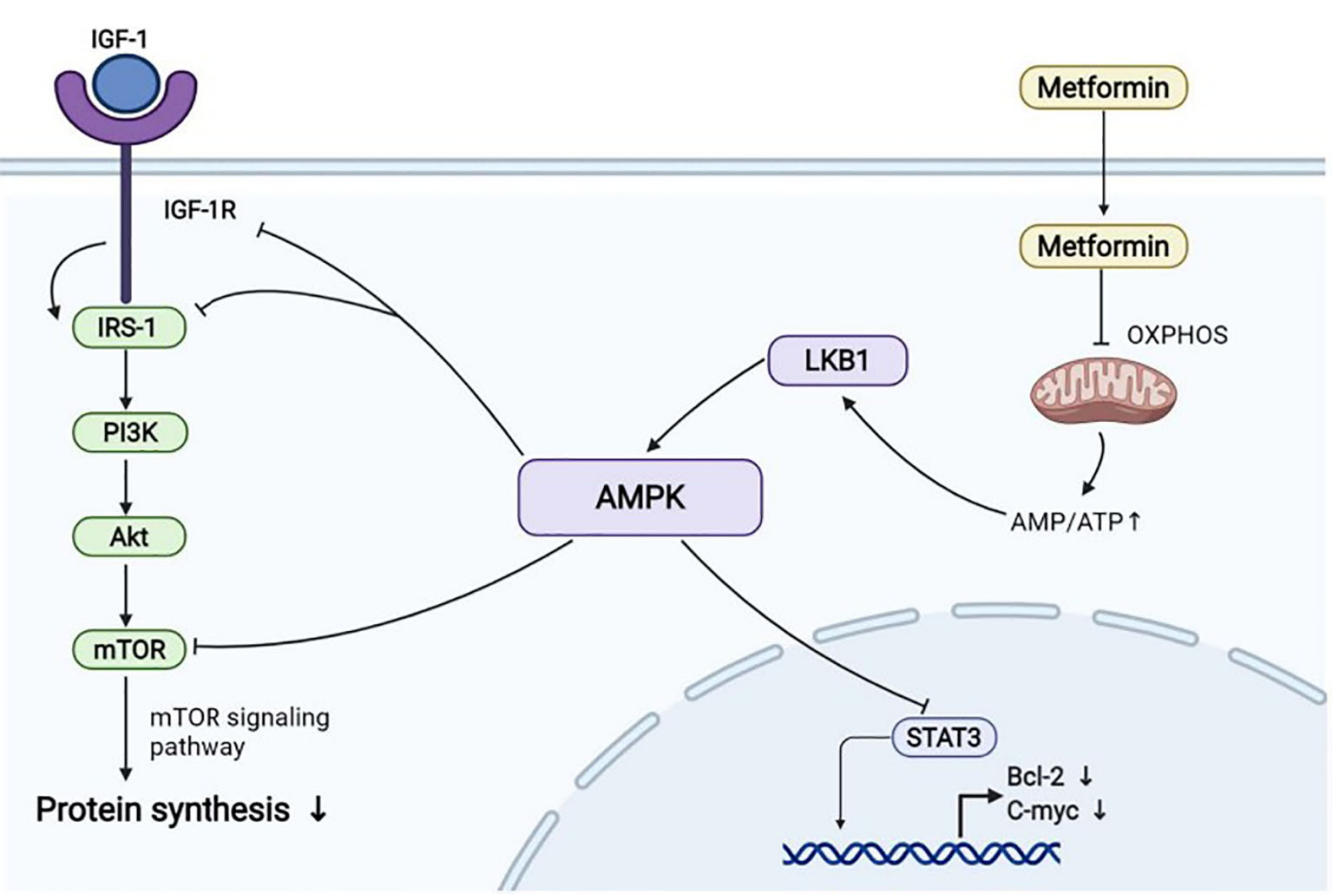
Some of the molecular mechanisms by which metformin inhibits cancer. (Created from BioRender.com.).

For obesity, there have been new therapeutic strategies for cancers induced by obesity-related diseases. Multiple studies have shown that metformin can be used as an adjunctive treatment for BC, EC, OC, and other cancers of the female reproductive system, thereby reducing the morbidity and mortality (68, 127–129) (**Figure 2**). By inhibiting oxidative phosphorylation at the mitochondrial level (OXPHOS), it increases the ratio of adenosine monophosphate (AMP) acid to adenosine triphosphate (ATP) (127), thus activating AMPK and inhibiting several key signaling pathways. For example, activated AMPK inhibits the mTOR signaling pathway and reduces the synthesis of tumor-related proteins (127, 128), thus inhibiting the progression of cancer. It also inhibits IGF-1/IRS-1/PI3K/AKT pathway. It can also lead to the activation of the cellular energy-sensing liver kinase B1 (LKB1)/AMPK pathway. LKB1, as a tumor suppressor, has a negative effect on the occurrence of many tumors (129). In addition, the transcription factor signal converter and transcriptional activator 3 (STAT3) are highly expressed in BC and EC. Metformin can block the phosphorylation of STAT3 and reduce the expression of c-myc, Bcl-2, and other growth promoting targets to inhibit the occurrence and development of these two cancers (127, 128). Therefore, metformin can be used as an effective drug for the treatment of MPCs associated with breast and gynecological cancers, providing a new idea for the treatment of BC-associated MPCs.

In addition to genetic screening and drug therapy, patients should also actively control their weight and quit smoking in time. Young women with BC need additional attention and research.

Otherwise, ibrutinib has been found to have various anti-tumor effects and has been shown to be effective in preclinical studies on OC, BC, and EC cell lines and animal models (130), which is undoubtedly a new treatment option for BC-associated MPCs.

The treatment of patients with MPCs associated with breast and gynecologic cancers is still mainly surgical treatment, supplemented by radiotherapy, chemotherapy, endocrine therapy, and other multidisciplinary comprehensive therapy. Among them, hormone receptor-positive patients should receive endocrine therapy, and through timely, accurate, and suitable treatment, most patients can improve their prognosis and life.

## Prognosis

The prognosis of MPCs associated with BC is worse than that of BC alone. The 5-year overall survival rate of BC patients with other primary cancers is about 4.7% lower than that of patients with BC alone (25).

It is worth mentioning that the study by Zhang et al. showed that the 5-year and 10-year overall survival rates of patients with BC after OC were 81.7% and 67.4%, respectively. However, the corresponding overall survival rates of patients with OC without subsequent malignant tumors were 17.0% and 6.5%, respectively (47), which were much lower than those of patients with double primary cancers. This indicates that the prognosis of MPCs is not mainly determined by the number of primary cancers, but by factors such as the patient’s age, cancer stage, pathological type, and malignant degree of the tumor. Individual differences among patients are vast, so the treatment process should be specialized according to the characteristics of different MPCs and patients in order to prolong survival and improve the quality of life.

## Discussion

The mammary gland is closely related to the female reproductive system in many aspects, affecting the whole gynecological system. The most common sites of MPCs associated with BC and gynecologic malignancies are the ovary, endometrium, and cervix. Although the incidence of BC associated with gynecologic malignancies is gradually increasing and more studies have been conducted in recent years, further research is required for more insights. The pathogenesis of MPCs is complex and not fully understood. It is related to the patient’s own susceptibility and unhealthy lifestyle, autoimmune deficiency, genetic causes, carcinogenic factors in the surrounding environment, treatment methods such as radiotherapy and chemotherapy. While risk factors such as smoking, alcohol consumption and unhealthy lifestyles can be eliminated, there are uncontrollable and unmodifiable risk factors such as genetic susceptibility and sometimes irreversible effects of anti-cancer treatments.

However, MPCs are often confused with metastasis or recurrence of malignant tumors in clinical practice, and secondary tumors of MPCs are of different clonal origin from primary tumors, while metastatic cancers are the same (123). However, the genetic material and biological behavior of tumor cells may be further changed due to the difference in local microenvironment during the colonization and proliferation of metastases in new sites, resulting in significant differences between their phenotypes and primary sites, which may lead to metastatic cancers misdiagnosis as MPCs, and for MPCs, mutations of selected gene sites may be the same because of tumor homogeneity or the same carcinogen, leading to misdiagnosis as metastatic cancer (131). If clinicians a lack of understanding of MPCs, will be the second primary cancer misdiagnosed as metastatic cancer, treatment strategies are set from the angle of the first primary cancer, and cell biology characteristics of various tumors in MPCs are independent and each are not identical, the treatment methods of different from metastatic carcinoma, so as to delay the best treatment time. Therefore, it is very important to clarify the characteristics of each tumor lesion for the diagnosis and treatment of patients with MPCs.

In addition to the difficulty in distinguishing MPCs from metastatic cancers, there is no unified treatment plan at present due to the complexity of pathogenesis and involved tumors. Some scholars believe that surgical treatment should be performed as soon as possible even if the second primary cancer has surgical characteristics (131). However, because MPCs associated with breast and gynecologic cancer exist in different organs and sites, their histological types are different, the reasons affecting treatment and prognosis are more complex, and patients’ physical and psychological conditions are worse than those of patients with single cancer, surgical treatment may limit the improvement of such patients’ conditions (99). Therefore, radical treatment is mainly used for different primary cancers, and the relevant treatment strategy should also be formulated according to the relevant primary cancer.

Therefore, in future studies, we need to focus on the pathogenesis of MPCs associated with breast and gynecologic cancers, reduce the toxic and side effects of various therapeutic measures, and carefully distinguish MPCs from metastatic cancers. Since there are essential differences in the cloning sources and development of the two, and there are pathogenic genes in patients with MPCs that lead to the occurrence of subsequent cancers, it may be a breakthrough for research to distinguish MPCs from metastatic cancers at the gene level.

For treatment of MPCs, multidisciplinary teams should focus on all risk factors and provide the most adequate management options. Early treatment should be given to patients to reduce the occurrence of MPCs from the source. When MPCs occur, the corresponding treatment plan should be formulated according to the severity of the patient’s disease, and reasonable personalized treatment plan should be provided for the patient. Considering heterogeneity and bias, further studies are needed to analyze clinical prognostic factors in order to establish a staging system for predicting prognosis.

## Conclusion

In conclusion, BC associated with OC, BC associated with EC, BC associated with CC are the common types of MPCs associated with breast and gynecologic cancers. Their risk factors include the presence of co-pathogenic genes, abnormal hormone levels, poor lifestyle and behavior habits, age, and treatment regimens of the first primary cancer. The pathogenesis of MPCs is complex and not fully understood and the prognosis of MPCs associated with BC is worse than that of BC alone. In addition, misdiagnosis of MPCs lead to the implementation of the wrong treatment plan and condition assessment, which will adversely impact the treatment of patients. Therefore, postoperative follow-up should be strengthened, various examinations should be improved, preoperative biopsy should be performed, pathological types should be identified, and correct treatment should be given in time. Through the understanding and research of the specific situation of patients with different types of MPCs associated with breast and gynecologic cancers, the corresponding treatment plan should be formulated, so as to achieve the purpose of treating the disease and improving the survival and quality of life of patients.

## Author Contributions

Conceptualization SG and XM validation XM writing—original draft preparation SG BW writing—review and editing SG BW ZW JH supervision XM All authors have read and agreed to the published version of the manuscript.

## Funding

This review was supported by the National Natural Science Foundation of China (No. 81872123); “Xingliao Talents Program” of Liaoning Province (No.XLYC1902003); Liaoning Provincial Higher Education Innovation Team; Distinguished Professor of Liaoning Province; China Medical University’s 2018 Discipline Construction “Major Special Construction Plan” (No. 3110118029);and Outstanding Scientific Fund of Shengjing Hospital (No. 201601).

## Conflict of Interest

The authors declare that the research was conducted in the absence of any commercial or financial relationships that could be construed as a potential conflict of interest.

## Publisher’s Note

All claims expressed in this article are solely those of the authors and do not necessarily represent those of their affiliated organizations, or those of the publisher, the editors and the reviewers. Any product that may be evaluated in this article, or claim that may be made by its manufacturer, is not guaranteed or endorsed by the publisher.
